# How to Get Rid of the Belief Bias: Boosting Analytical Thinking via Pragmatics

**DOI:** 10.5964/ejop.v15i3.1794

**Published:** 2019-09-27

**Authors:** Laura Macchi, Francesco Poli, Laura Caravona, Michela Vezzoli, Miriam A. G. Franchella, Maria Bagassi

**Affiliations:** a Department of Psychology, University of Milano-Bicocca, Milan, Italy; b Donders Institute for Brain, Cognition and Behaviour, Radboud University, Nijmegen, The Netherlands; c Department of Historical Studies, University of Milan, Milan, Italy; Trinity College Dublin, Dublin, Ireland

**Keywords:** belief bias, categorical syllogism, pragmatics, analytical thinking, dual-process theories

## Abstract

The previous research attempts to reduce the influence of the belief bias on deductive thinking have often been unsuccessful and, when they succeeded, they failed to replicate. In this paper, we propose a new way to see an old problem. Instead of considering the analytical abilities of the respondent, we focus on the communicative characteristics of the experimental task. By changing the pragmatics into play through a subtle manipulation of the instruction of the syllogism problem, we obtained a strong improvement in the accuracy of the performance in both untrained and trained in logic respondents. We suggest that current models of deductive thinking should be broadened to consider also communicative understanding as part of the processing of the problem.

Deductive reasoning is pervasive and commonly used in everyday life. It allows, starting from some premises, to establish one (or more) conclusions that may do not extend our knowledge, but which are necessarily true. Therefore, it allows us to establish the consequences of our beliefs. If all my friends greet me when they meet me (*Premise 1*) and John is a friend of mine (*Premise 2*), then I expect John to greet me when he meets me (*Conclusion*). This kind of syllogism allows us to build expectations on how reality should occur and, thus, helps us to guide our behavior.

Besides being a common type of reasoning, deduction is also part of logic. In logic it is possible to establish with certainty whether a deductive reasoning is valid or invalid through a propositional calculus. Psychology borrowed the propositional calculus to fully describe human reasoning, presupposing that it consisted *sic et simpliciter* with a formal logical system (Henle, 1962; Piaget, 1953). In this sense, therefore, logic was used as a psychological descriptive theory of human thought. But soon it became clear that, although under certain conditions the human being was able to reason according to the criteria of logic, under other specific and systematic conditions s/he produced errors of reasoning, called biases.

An emblematic example of such biases is the belief bias that can be defined as the systematic tendency to evaluate a syllogism through the credibility of its conclusion rather than through the principle of logical necessity (Evans, Barston, & Pollard, 1983; Evans & Curtis-Holmes, 2005). The belief bias is one of the first reasoning biases ever discovered (Morgan & Morton, 1944; Wilkins, 1929) and has resisted stricter methodological controls over the years (Barston, 1986; De Neys & Franssens, 2009; Evans et al., 1983; Evans, Handley, & Harper, 2001; Evans, Newstead, & Byrne, 1993; Johnson-Laird & Byrne, 1991; Markovits & Nantel, 1989; Roberts & Sykes, 2003; Shynkaruk & Thompson, 2006).

Traditionally, the belief bias has been studied using categorical syllogisms that are deductive reasoning problems composed of two premises and a conclusion. Studies using categorical syllogisms could involve a conclusion-evaluation or a conclusion-generation paradigm. The former requires participants to evaluate the validity of a conclusion that is presented, while in the latter participants are asked to generate a logical conclusion in response to given premises. Most of the studies on the belief bias focused on the conclusion-evaluation paradigm, and therefore the following discussion centers on findings deriving from this paradigm. Yet, it must be recognized that any comprehensive theory of belief bias should also capture data acquired with conclusion-generation tasks.

The following examples (Ball & Stupple, 2016) depict how logical validity and conclusion credibility are usually manipulated in studies on the belief bias:

(1) Valid-Believable syllogism:

All birds have wings

All crows are birds

Therefore, all crows have wings

(2) Valid-Unbelievable syllogism:

All mammals can walk

All whales are mammals

Therefore, all whales can walk

(3) Invalid-Believable syllogism:

All flowers need water

All roses need water

Therefore, all roses are flowers

(4) Invalid-Unbelievable syllogism:

All meat products can be eaten

All apples can be eaten

Therefore, all apples are meat products

The common findings in the literature show that most people consider (1) as valid, but not (2). However, they share the same logical structure; thus, they are both valid. Moreover, they consider (3) as valid, but it is not. Therefore, the credibility of the conclusion affects the conclusion’s validity evaluation: syllogisms with the same structure are considered as valid or invalid depending on whether the content of the conclusion is believable or not. Typically, the validity judgment given by the participants departs from the logical validity in two cases: when the syllogism is logically invalid, but the content makes it believable (Invalid-Believable) and when the syllogism is logically valid, but the content makes it unbelievable (Valid-Unbelievable).

To explain this type of bias, dual-process theories of thinking have been put forward. They are characterized by the distinction between two different systems of reasoning: an associative system, which is fast, automatic, unconscious and effortless (System 1), and a rule-based system, which is slow, serial, conscious and effortful (System 2). According to the theory, reasoning errors such as the belief bias are caused by the tendency to rely on System 1, while the involvement of System 2 would allow avoiding reasoning errors and would guarantee correct reasoning.

The general term “dual-process theories” encompasses a wide number of models, which can be divided into different classes. A first class is composed of models that theorize a staged, sequential progression from System 1 to System 2, such as the Selective-Scrutiny model espoused by Evans et al. (1983; for its more recent instantiation, termed selective-processing model, see Evans, 2007, 2008; Evans & Stanovich, 2013) or the Mental Models Theory proposed by Oakhill, Johnson-Laird, and Garnham (1989; see also Johnson-Laird & Byrne, 1991). This class of theories shares the idea that unbelievable conclusions trigger analytic processing.

A second class of models is named parallel-process account (Sloman, 1996; Stupple & Ball, 2008), as it is founded on the assumption that System 1 and System 2 operate concurrently.

A further model that is worth mentioning is the Misinterpreted-Necessity model (Barston, 1986) which argues that when solving syllogisms, human beings misinterpret the logical meaning of necessity. Hence, in some instances, this misunderstanding leads to a belief-based decision.

Independently of which specific model is preferred, all of them are characterized by the presence of a System 2 that can abstract information from the context, thus producing unbiased solutions (cognitive decoupling) and by a System 1 that, instead, automatically contextualizes the information and thus bases its response predominantly on the content of the syllogism, not on its logical validity. Hence, according to the dualistic approach, the error could be attributed to a lack of decontextualization, which is caused by a strict reliance on System 1 and by the failure to employ System 2 (Evans & Stanovich, 2013; Stanovich & West, 1999, 2008).

The most compelling evidence that links the reliance on System 2 to a better performance in belief-biased problems have been obtained by correlating general cognitive abilities with the ability to solve problems containing biasing contextual information. Sá, West, and Stanovich (1999) found that relatively higher cognitive ability (measured with SAT), greater disposition toward active and open-minded thinking and greater ability to decontextualize are all associated with a reduced probability that the response to belief-biased syllogisms is based on prior beliefs (Stanovich, West, & Toplak, 2016).

Dualistic theories are undoubtedly powerful in describing how the human mind works when it must tackle a logical problem. Consequently, they also allow to formulate a prediction on how we can improve the performance in belief-biased problems: First, since System 2 seems to be initially inactive (or at least, less active than expected), it should be somehow triggered. Additionally, one would ideally aim at inhibiting the automatic reliance on System 1. To the best of our knowledge, all the previous studies that attempted to improve performance in belief-biased tasks were based on those assumptions. So far, it has been hard to overcome the bias: Experimental manipulations always resulted in absent or weak effects (Evans, Newstead, Allen, & Pollard, 1994; Evans et al., 2001; Handley, Newstead, & Trippas, 2011). In our view, even if triggering more heavily the analytical abilities that a person possesses is an important step to improve performance, its role has been overrated in previous research.

An example that may help us to understand which factors trigger analytical reasoning can be found in Stupple, Ball, Evans, and Kamal-Smith (2011). The authors divided participants in 3 groups depending on their performance on belief-biased syllogisms, obtaining high-performance, medium-performance, and low-performance groups. Then, they analyzed participants’ response times to the syllogisms and found that individuals that were performing worse (low-performance group) were also quicker in solving them, compared to individuals performing well. Since analytic thinking is slower than heuristic thinking, the slower response times of the high-performance individuals were interpreted by the authors as an index of the greater reliance on the analytic system. Hence, they concluded that individuals that are more able to avoid the belief bias are the ones that *possess* higher logical abilities. However, this interpretation is all but compelling, as the only thing that is reasonable to conclude is that individuals who are more able to avoid the bias are the ones that *engage more* in analytical thinking, but this does not explain *why* they do so. It may be that they possess greater logical abilities than others, but there may be other reasons. It could be that they have better understood the problem, and thus they know they should use analytical skills. Consequently, they engage more in analytical thinking, but this does not mean they possess greater analytical skills. Even the greatest analytical mind may fail to solve the problem if s/he does not understand that thinking analytically is required in that specific situation.

Indeed, we argue that, before any system (heuristic or analytic) is involved, another fundamental step in the processing of the problem must be considered: the comprehension of the task’s aim. In other words, are we sure that a person that engages in a belief-biased task understands and represents the task in the same way as the experimenter has represented it? This communicative aspect has often been ignored in the previous studies on the belief bias, and it will be analyzed in greater detail in the next chapter.

However, it is important to stress that the above-mentioned study by Stupple et al. (2011) brought up a further, crucial piece of evidence: Individuals tended to answer more quickly in no-conflict situations (i.e., when confronted with a Valid-Believable or an Invalid-Unbelievable syllogism) compared to conflict situations (i.e., when dealing with Invalid-Believable or Valid-Unbelievable syllogisms). This effect was particularly evident for the high-performance group. However, even in the low-performance group appeared the same pattern, suggesting that all individuals, at some point in the processing of the problem, were very likely unconsciously—detecting the conflict between logical thinking and knowledge of the world. This finding is very intriguing for multiple reasons (see Ball & Stupple, 2016) but particularly because it suggests that individuals might be able to think logically, if only they were adequately triggered to do so. As De Neys (2012) puts it, people are in some way able to “detect that they are biased” (p. 29).

Can we activate this detection process? Once the conflict is detected, how can we inhibit the response based on fast processing (knowledge of the world), in favor of the slower analytical processing? We successfully managed to do so, thus improving the response accuracy in belief-biased syllogisms, using a pragmatic approach to language and thinking.

## The Pragmatic Approach

According to us, everything that the text of the considered problems communicates affects the representation of the decisional framework: not only what literally is said (the sentence), but also what is implied and then effectively communicated. The distinction between sentence and utterance is the core of Grice’s communication theory (Grice, 1975), according to which the phrases imply and mean more than what they literally state. What is implied is the outcome of an inferential process, in which what is said is interpreted in the light of the intentions attributed to the speaker and of the context unavoidably elicited and determined by any communications.

The central idea is that communication is achieved when a recipient recognizes the special kind of intention with which a communicative act is produced. More generally, Grice’s theory of implicature postulates that meaning should be reduced to intention and, therefore, that semantics has to be reduced to psychology.

We consider communication and thinking as two sides of the same cognitive process, which realizes in the discourse in the same way as the ancient Greeks intended “logos”. In Greek, in fact, the word “logos” means at the same time “language”, but also “calculation”, “reasoning”. Any time we use language, we are not just “transmitting thinking” (Chomsky, 1977, p. 90), but we are actually thinking. More precisely, what we mean is that “discourse-thinking” is a human cognitive process (Mosconi, 1990, 2016). Accordingly, analysis of the discourse is the proper methodology for studying reasoning.

The intuition that the rules of discourse influence the inferential processes does not seem surprising. Problems studied in reasoning, indeed, are mainly submitted in a verbal form. Nevertheless, this obvious intuition did not influence the psychology of thinking and reasoning, at least until a few years ago.

Numerous studies have shown the importance of the pragmatic approach to the study of thinking and reasoning (Bagassi & Macchi, 2016; Hilton, 1995; Politzer & Macchi, 2000) from problem-solving (Macchi & Bagassi, 2012, 2015), conditional reasoning (Sperber, Cara, & Girotto, 1995) and deductive reasoning (Van Der Henst, Sperber, & Politzer, 2002) to probabilistic reasoning (Dulany & Hilton, 1991; Macchi, 1995, 2000; Mosconi & Macchi, 2001; Politzer & Macchi, 2005), decision making (Bagassi & Macchi, 2006) and children’s reasoning (Bagassi, D’Addario, Macchi, & Sala, 2009).

Consider, as an example, the results obtained by Tversky and Kahneman (1983) with the well-known “Linda Problem.” Not only laypeople but also informed and even sophisticated subjects in the field of statistics committed the so-called conjunction fallacy, failing to recognize the conjunction rule (i.e., the probability of two events occurring together is always less than or equal to the probability of either one occurring alone). In our view, the fact that people trained in decoupling failed to recognize the inclusion rule, which is a relatively elementary logical rule, raises questions that need to be addressed (Macchi & Bagassi, 2018). As stressed by Bagassi and Macchi (2016; see also Dulany & Hilton, 1991; Mosconi & Macchi, 2001), the misleading contextualization of the task, which was centered on an irrelevant personality sketch from a logical perspective, hindered even statistically trained respondents from grasping the experimenter’s communicative aim concerning the inclusion class rule.

Analogously, in a recent experiment involving the Wason Selection Task, we found (Bagassi & Macchi, 2016) that the performance of participants trained in logic did not differ significantly to that of untrained participants who had not received such training. With the original Wason Selection Task, only 13% of the trained respondents and 5% of the untrained group gave the correct response. Paradoxically, a great number of participants were able to use the material implication correctly but were not able to identify the only case that was crucial for falsifying the rule. How can it be that people trained in logic or statistics had such difficulty with these tasks? One would have expected that their training and experience would have facilitated them in logical reasoning, but this was not the case.

A similar issue can be raised when considering Invalid-Believable syllogisms such as the following, which are very common in the literature:

All flowers fade

All roses fade

All roses are flowers

Advocates of dualistic theories of thought would argue that people will accept the invalid conclusion because when there is a conflict between logic and beliefs, beliefs always prevail. But, as observed by Bagassi and Macchi (2016) the terms “roses” and “flowers” can be seen as two classes, and crucially the former is implicitly included in the latter. Hence, one can implicitly infer that “all roses are flowers” (which is the conclusion of the Invalid-Believable syllogism), but according to logic it is not correct from the two premises to conclude that “roses are flowers.”

However, this error from a logical point of view can be interpreted as the result of a cooperative act: concluding that roses are flowers does not add knowledge, it only allows the communicative interaction, although at the lowest level of informativeness. As in the Socratic dialogue, rhetorical questions have a common basic function from which the argument will start and will be developed. Saying that “roses are flowers” is to give its own consent on an obvious but shared knowledge. In a non-experimental context the two premises would raise a spontaneous reply such as “We know this, so what?”, the natural equivalent to the logical: nothing ensues (Bagassi, & Macchi, 2016; p. 49).

A further example of the importance of a correct understanding of the aim of the experimenter can be found in a study by Mata, Schubert and Ferreira (2014; see also, Mata, Ferreira, Voss, & Kollei, 2017). They asked participants to solve many different problems, among which there were syllogisms. Crucially, syllogisms had a conflict and a no-conflict version. In the conflict version, the syllogism was invalid but believable:

All flowers need water

Roses need water

Roses are flowers

In the no-conflict version, the syllogism was valid and believable:

All flowers need water

All roses are flowers

Roses need water

Even if the first premise is identical, the two syllogisms differ in the second premise and in the conclusion. Mata et al. (2014) found that participants that were more able to detect the differences between the two versions were also more likely to solve the conflict version correctly.

In our view, a successful representation of the aim of the problem (i.e., what the experimenter wants the participants to do) seems to be crucial for a successful performance. We speculate that the few who answered correctly to the belief-biased syllogisms had a particular ability to grasp the intention of the experimenter and the aim of the task rather than the ability to decouple from contexts and contents. In so doing, they revealed a form of “interactional intelligence” and “interpretative ability” (Bagassi & Macchi, 2016).

How do participants reach an understanding of the experimenter’s intentions? Crucially, within a pragmatic framework (Grice, 1957, 1975; Levinson, 1983; Mosconi, 1990, 2016; Sperber & Wilson, 1986) communication is not limited to what is said (i.e., the content that the experimenter intends to communicate explicitly to a participant), but it also involves what is implied (i.e., the content that the experimenter intends to communicate implicitly using that particular expression). Therefore, there is an implicit content that participants must infer through a specific type of conversational implicature. We believe that only a few individuals that possess a high interactional intelligence manage to do so, thus inferring the experimenter’s intentions.

This crucial feature of communicative interactions has two important consequences for the topic dealt with here.

In general, in experimental psychology, communicative interactions between experimenters and participants unavoidably occur. These communicative interactions are not exempt from implicit pragmatic inferences, and it is therefore extremely important to evaluate in detail the implicit content that one is communicating using a specific instruction or a specific statement.

Specifically, in psychology of thought it is necessary to stress that natural language and logical language do not possess the same type of rules (nor even the same type of rule violation, see Bagassi & Macchi, 2006; Hilton, 1995; Levinson, 1983, 2000; Mosconi, 2016; Politzer & Macchi, 2000). Both logic and natural language have the common goal of transmitting meaning efficaciously. However, as suggested by Bagassi and Macchi (2016), this objective is achieved by these two language forms in opposite ways.

Logic achieves a univocal communication through simplification, eliminating any meaning that might interfere with the univocal meaning to be communicated. On the contrary, natural language exploits the expressive richness of the words but avoids slipping into chaos and tripping over misunderstandings, by relying on the pertinence of the meaning to the context. There is no hierarchical order between natural language and logical language in the sense that the former is inferior or subordinate to the latter. The two reflect different needs: in the first case, the need to ensure the efficacy of the communication; in the second, the need to guarantee the rigor of the inferential process (Bagassi & Macchi, 2016, p. 46).

In general, even if participants are required to infer the experimenter’s intentions, experimenters have communicative obligations too. They are required to be cooperative (Grice, 1975) and to express what they mean clearly and adequately to avoid misunderstanding. In previous literature, the instructions utilized by experimenters did not communicate the aim of the task optimally. In fact, before reading the syllogism, the participant usually finds a long text in which s/he is recommended to think logically. For example, Evans et al. (1983, Experiment 1) gave to participants long instructions that, according to us, contain conflicting information. The participant was asked to think logically (“you will be given a prose passage to read and asked if a certain conclusion may be logically deduced from it”), and at the same time to assume that all information was true (“you should answer this question on the assumption that all the information given in the passage is, in fact, true”). To untrained participants, the boundary between truth and validity might be fuzzy, and their co-presence in the instructions might facilitate the confusion between the two. Thus, the participant may end up implying that they are the same thing and that s/he is allowed to think just on the believability of the content. This is in line with the misinterpreted-necessity hypothesis (Barston, 1986), which argues that incorrect performance originates from a misunderstanding of logical necessity.

Hence, in later research attempts have been made to improve performance in belief bias through the manipulation of task instructions, emphasizing that only logically-necessary conclusions must be drawn (Barston, 1986; Evans et al., 2001; Handley et al., 2011) but they were not successful.

Newstead, Pollard, Evans, and Allen (1992, Experiment 5) obtained an improvement with very long instructions in which it was explained how logic works and what is meant by logical validity and necessity. The improvement in performance was remarkable, as 83% of the participants responded correctly with invalid-believable syllogisms. We do not deny that their results may be partly due to the continuous stressing of the importance of logical validity, but other factors may be at play. In fact, their instructions stressed that if the participant was not “absolutely sure” about the validity of the conclusion, s/he must had rejected it. This may have triggered a general tendency toward rejection. Consistently, acceptance rates for valid-believable syllogisms were lower than usual (75%, against the 92% of the study by Evans et al., 1983) implying a paradoxical worsening of the performance. Furthermore, they obtained remarkably high rejection rates (50%) even in the control condition, as if they picked up a particularly talented sample. Consistently with these observations, an attempt to replicate the results (Evans et al., 1994) was less successful. The same instructions led to poorer performance in the invalid-believable syllogisms (56% of correct responses), and the acceptance of valid-believable syllogisms was still slightly lower than usual (81%). See Table 1 for comparing the cited results.

**Table 1:** Percentages of Correct Responses for Valid-Believable and Invalid-Believable Syllogisms in the Three Different Studies.

Experiment	Valid-believable	Invalid-believable
Evans et al. 1983	92%	8%
Newstead et al. 1992 (Exp. 5)	75%	83%
Evans et al. 1994	81%	56%

*Note.* A correct response in valid-believable syllogisms implies the acceptance of the conclusion, while a correct response in invalid-believable syllogisms implies the rejection of the conclusion.

Hence, in previous attempts to improve performance, instructions were probably closer to the task aim than the original one, but still inadequate. The key point we would like to stress is that for an instruction manipulation to be successful, it must follow pragmatic principles and maxims (Grice, 1957; Levinson, 1983, 2000), and such characteristic was absent in previous research.

## Experimental Research

From the theoretical assumptions described above, it is possible to draw the following experimental hypotheses:

1. When the instructions do not clearly convey the exact nature of evaluation of the inference, and thus it is not possible for the participant to understand how s/he is supposed to reason (following the rules of natural language and common knowledge of the world or following the rules of logic), the strength of the belief bias will be unrelated to the degree of expertise in logic. In other words, not even people that have been trained in logic can solve the problem, if they cannot understand that they must think logically. Hence, in experimental terms, we expect that the degree of acceptance of invalid-believable syllogisms (which is an index of the strength of the belief bias) will be high both in respondents trained in logic and in respondents that are untrained to logic.
2. Subtle modifications of the instructions can lead to strong changes in performance, if such modifications are aimed specifically at making clearer the aim of the task. The experimenter must make the participant understand that s/he is not expected to follow the rules that are commonly present in the natural language (i.e., reasoning taking into account truth), but that s/he has to switch to different rules, the ones of logical language (i.e., reasoning considering necessity): as demonstrated by Bagassi and Macchi (2016), the performance of participants trained in logic did not differ significantly to that of untrained participants who had not received such training. How can it be that people trained in logic had such difficulty with these tasks? One would have expected that their training would have facilitated them in decontextualizing and abstracting, but that was not the case. Paradoxically participants that were able to execute the material implication truth table correctly were not able to identify the only case that was crucial for falsifying the rule. In the absence of specific indications, they reasoned in the same manner as the untrained group and used conversational rules of natural language to interpret.

## Experiment 1

Experiment 1 was designed to test the first experimental hypothesis according to which the performance of participants trained in logic do not differ from that of untrained participants who had not received such training.

### Method

#### Participants and Procedure

The first experiment involved 130 undergraduate students from humanity faculties of Milano-Bicocca University. Participants included males and females and were aged between 19 and 25 years. In the sample, there are 50 untrained and 80 trained in logic participants. Participants were considered as trained in logic if they followed at least one 64-hour university annual course concerning fundamentals of logic and took the exam. The day of the exam, some additional sheets were given to students together with the exam sheets. Students were invited to fill in the additional sheets at the end of the exam and it was made clear that they concerned an experiment on psychology of thought. Untrained participants were university students that didn’t follow any course on logic, and they were asked to participate to the experiment at the end of a lesson.

#### Material

In this experiment we used the conclusion-evaluation paradigm that is evaluating the validity of the given conclusion based on the premises. We asked to each participant to perform a paper-and-pencil task containing two different syllogisms presented in random order. One syllogism was valid-believable:

All animals are mortal

All humans are animals

All humans are mortal

The other syllogism was invalid-believable:

All flowers fade

All roses fade

All roses are flowers

Both syllogisms were followed by the instruction: “Does the conclusion follow from the premises?” The instruction was followed by the question “Why?” that allowed us to gather additional qualitative information on the conscious cognitive processes performed by the participants.

### Results

**Figure 1:**
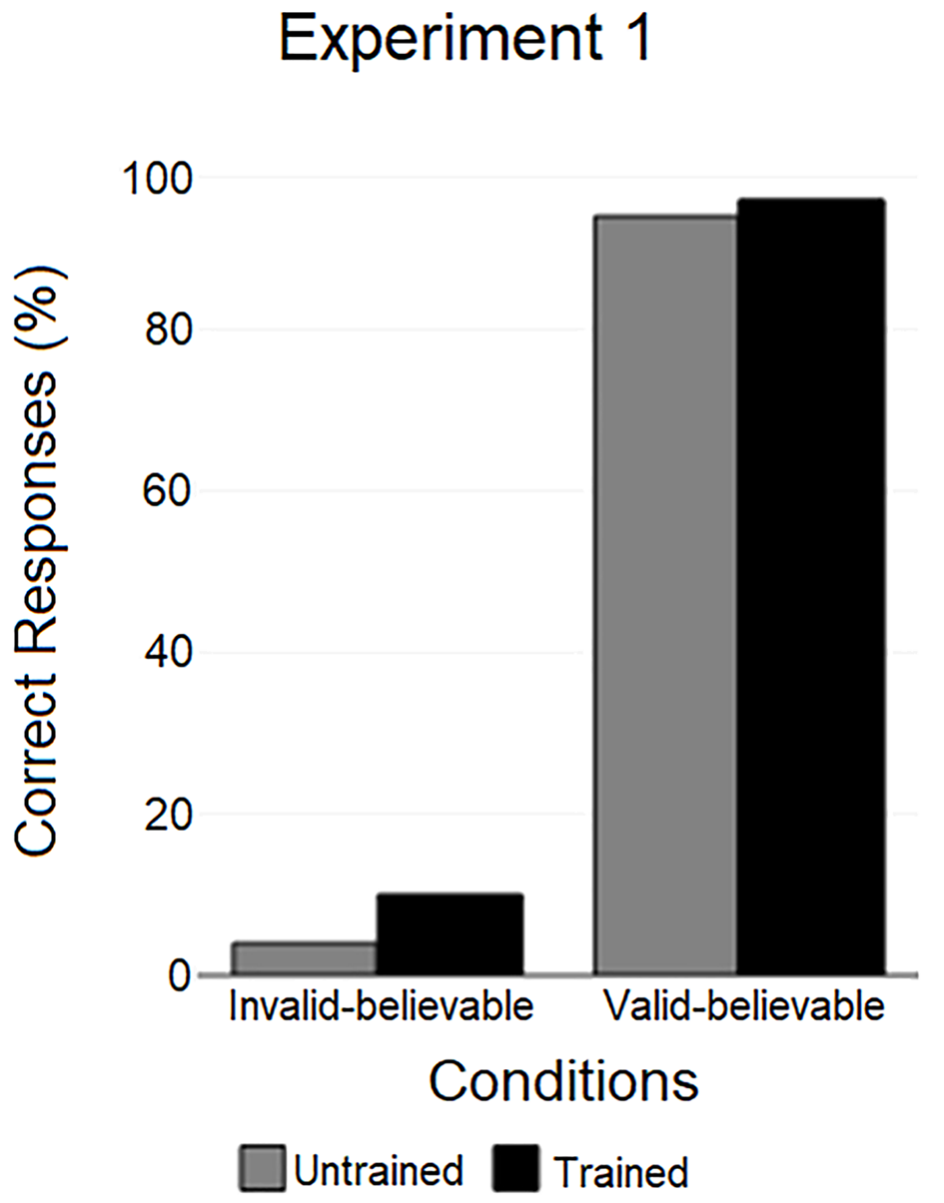
Percentage of correct responses for the valid-believable syllogism and for the invalid-believable syllogism in untrained and trained participants.

A highly significant difference was found between the performance in the valid-believable syllogism and the invalid-believable syllogism, both in untrained (χ^2^(2, *N* = 50) = 81.03, *p* < .001) and trained participants (χ^2^(2, *N* = 80) = 119.49, *p* < .001). In the valid-believable syllogism, 94% of untrained participants and 96% of trained participants responded correctly (see Figure 1) and there was no statistical difference between the two groups (χ^2^(2, *N* = 130) = 0.95, *p* = .69); In the invalid-believable syllogism, 4% of untrained participants and 10% of trained participants responded correctly and performance did not differ significantly between the two groups (χ^2^(2, *N* = 130) = 1.56, *p* = .31).

To assess the strength of evidence in favor of the hypotheses that there are no differences in the performance of the trained group and the untrained group, we used the Bayesian contingency table analysis. Statistical analyses were conducted using the free software JASP and default priors (JASP Team, 2018). Moreover, we used the fixed column multinomial sampling method (Jamil et al., 2017) because the number of participants in the untrained and logic groups were fixed. The Bayes factor suggests that, for the invalid-believable syllogism the hypothesis stating no differences between groups is 8.52 times more likely than the alternative hypothesis (95% CI: [−1.038, 2.016]); for the valid-believable syllogism, our hypothesis of independence is 4.20 times more likely than the dependence hypothesis (95% CI: [−0.504, 2.422]). This can be considered as moderate evidence in favor of our hypotheses (Lee & Wagenmakers, 2013).

### Discussion

Both with valid-believable and invalid-believable syllogisms, the performance of untrained participants was comparable to that of trained participants, thus suggesting that the degree of expertise in logic has no influence on the performance when it is not possible for the respondent to infer from the instructions that s/he is expected to think logically. Moreover, the performance was very high with the valid-believable syllogism, suggesting that the instructions allowed the participant to obtain a satisfactory performance in the no-conflict situation.

## Experiment 2

Experiment 2 was designed to test the second experimental hypothesis according to which the pragmatic manipulations of the instructions may change how the participants understand experimenter’s intentions and the aim of the task and thus improve the performance. Given that our goal was to observe an improvement in performance, we focused only on the invalid-believable syllogism, which is characterized by very low rates of correct responses.

### Method

#### Participants and Procedure

The second experiment involved 162 undergraduate students from humanity faculties of University of Milan-Bicocca. Participants included males and females and most were aged between 19 and 25 years; 101 participants were considered untrained and 61 trained in logic. Again, we considered as “trained” those participants that followed at least one 64-hour university annual course concerning fundamentals of logic and took the exam. The procedure was identical to that of Experiment 1. The control group considered in this experiment refers to the participants that worked on the invalid-believable syllogism in Experiment 1.

#### Material

One invalid-believable syllogism was administered to each participant (the same used in the first experiment):

All flowers fade

All roses fade

All roses are flowers

We manipulated the instruction of experiment 1 in two ways, with the intention of communicating to the participant to follow the principle of logical necessity, and with the subsequent expectation to see an improvement in the performance. The new instructions were: “Does the conclusion necessarily follow from the given premises?” (From now on, the *Necessarily* instruction) and “Can you infer, following the criteria of Logic, the conclusion from the given premises?” (From now on, the *Criteria-of-Logic* instruction). The two instructions are similar, but they stress different aspects. The former communicates directly the principle of logical necessity, stressing the type of relation that must occur between the premises and the conclusions, thus explaining the essence of logical, deductive thinking. The latter communicates, more indirectly, that the participant is expected to strictly adhere to logical rules while judging the validity of the syllogism. This reminds to a knowledge common to logicians, telling untrained respondents just that the type of reasoning is different from the usual one.

Hence, in this second case an improvement in the performance can be seen only if the participant has at least a rough idea of how logic works. For this reason, we can make a further prediction: The *Necessarily* instruction will lead to a similar improvement in performance both in untrained and trained participants, whereas the *Criteria-of-Logic* instruction will improve the performance of the trained group more than that of the untrained group, as they know better which are the logical criteria that must be followed.

For half of the participants the syllogism was followed by the *Necessarily* instruction and for the other half of the participants it was followed by the *Criteria-of-Logic* instruction. As in the first experiment, instructions were always followed by the question “Why?”.

### Results

When tested with the *Criteria-of-Logic* instruction, 41% of untrained participants and 67% of trained participants answered to the invalid-believable syllogism correctly. The difference between the two groups was statistically significant (χ^2^(2, *N* = 80) = 4.91, *p* = .03). When tested with the *Necessarily* instruction, 66% of untrained participants and 77% of trained participants answered correctly. As expected, there was no significant difference between the two groups (χ^2^(2, *N* = 82) = 1.02, *p* = .45).

**Figure 2:**
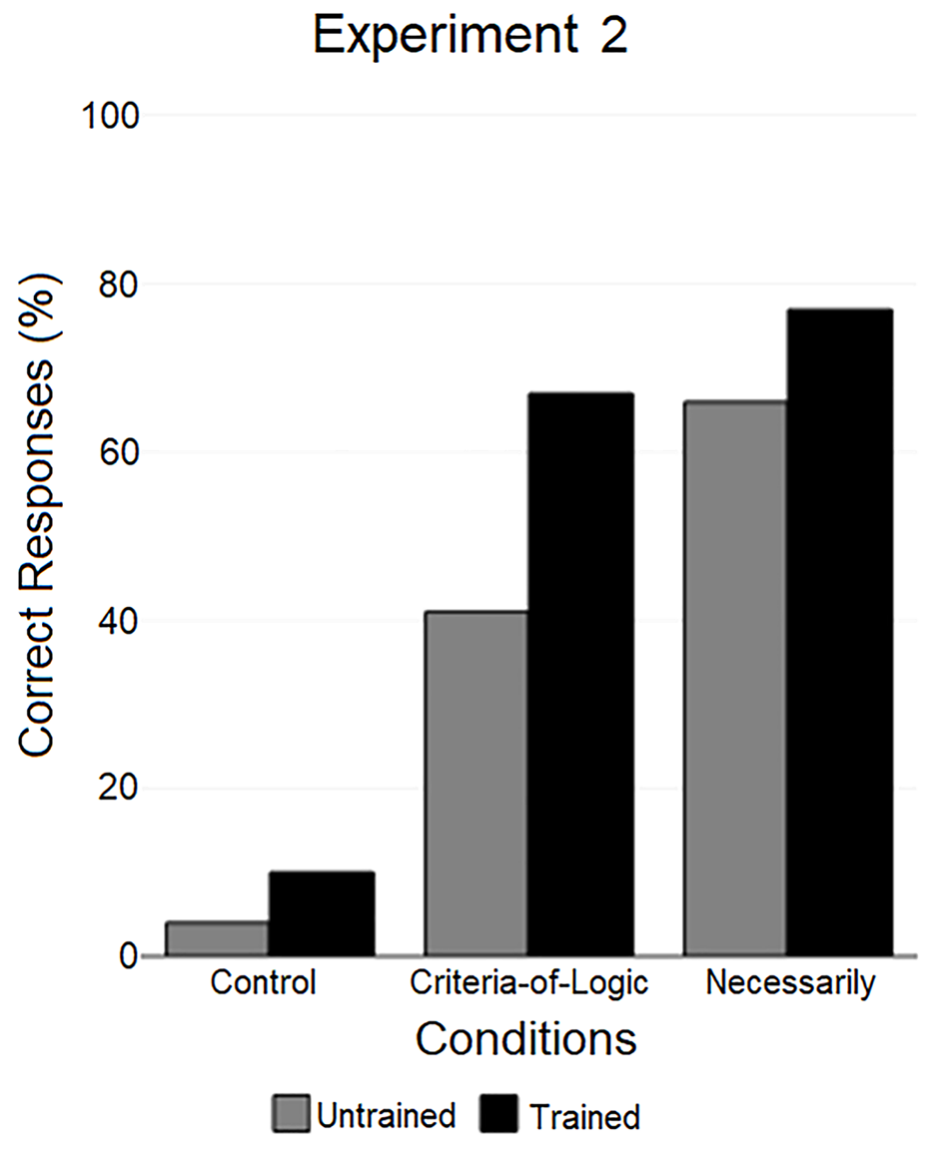
Results of experiment 2 (*criteria-of-logic* condition and *necessarily* condition) compared to the results obtained with the same syllogism in experiment 1 (control).

When comparing untrained participants’ performance with the *Criteria-of-Logic* instruction to untrained participants’ performance with the *Necessarily* instruction (see Figure 2), a significant difference was found (χ^2^(2, *N* = 101) = 6.25, *p* = .017). Instead, when comparing trained participants’ performance with the *Criteria-of-Logic* instruction to trained participants’ performance with the *Necessarily* instruction, the difference was not statistically significant (χ^2^(2, *N* = 61) = 0.74, *p* = .39).

Further comparisons were made between the results obtained in the control group and the results obtained with the same syllogism in the two experimental conditions of Experiment 2. The performance of untrained participants of the control group differed significantly from the performance of untrained participants in the experimental groups, both when compared to the *Criteria-of-Logic* condition (χ^2^(2, *N* = 100) = 19.84, *p* < .001) and the *Necessarily* condition (χ^2^(2, *N* = 101) = 42.24, *p* < .001). Similar results were found analyzing the performance of trained participants, as there was both a significant difference between the control condition and the *Criteria-of-Logic* condition (χ^2^(2, *N* = 110) = 36.92, *p* < .001) and between the control condition and the *Necessarily* condition (χ^2^(2, *N* = 111) = 47.91, *p* < .001).

### Discussion

The results of the experiment show that the manipulation of the instructions by using a pragmatic approach led to an improvement in the performance, both in untrained and trained participants. With the *Necessarily* instruction, which clearly and concisely stresses that there must be a relationship of logical necessity between the premises and the conclusion, performance was greatly improved independently from the level of expertise in logic of the respondent. A similar improvement was found with the *Criteria-of-Logic* instruction for the trained group. As expected, the same instruction improved also untrained participants’ performance, but the improvement was not as consistent as for the trained group.

## General Discussion

A consolidated line of research claims that the ability to abstract (cognitive decoupling) is an essential condition for correct reasoning. However, from a pragmatic point of view, it might be argued that the participant who performs syllogisms correctly, without falling into the belief bias, is not just required to possess an ability for abstraction. Indeed, s/he must possess what we can call “interpretative talent” (Bagassi & Macchi, 2016), a skill in perceiving the real aim of the task, better contextualizing the information rather than de-contextualizing it. Indeed, the person must be able to understand a) the communicative intention of the experimenter; and b) that what s/he is required to do is to shift from an interpretation of the task based on the communicative rules we commonly use (the ones of natural language), to a different interpretation based on a different linguistic tool, that is logic.

In line with this hypothesis, a number of interesting studies shows that beliefs affect both intuitive and reflective reasoning (Verschueren, Schaeken, & D’Ydewalle, 2005) and that many cognitive biases are independent of cognitive abilities (Evans, Handley, Neilens, & Over, 2007; Stanovich & West, 2008) . Moreover, the ability to understand another person’s intentions are often conceptualized as Theory of Mind, and in the literature, it is generally agreed that Theory of Mind is linked to executive functions (Carlson & Moses, 2001; Chasiotis, Kiessling, Campos, & Hofer, 2006; Setoh, Scott, & Baillargeon, 2016). Hence, even when a correlation between cognitive abilities and performance in belief-biased problems has been found, it was well possible that Theory of Mind was playing a role as mediating factor^1^, and the better performance could have also been partially explained by a greater interpretative talent. However, this is just a speculation that would be interesting to further investigate in future research.

In summary, we succeeded in improving the performance of participants that were untrained to logic, bringing them to the same level as participants who were trained in logic. The current results suggest that what in literature has been considered as a typical error of logical thinking is actually a consequence of a task that does not clarify that the requested layer of evaluation is different from the common sense, by distinguishing between factual truth and logical validity.

We improved the performance of untrained participants by triggering those analytical abilities that respondents already possess, but that they failed to express with the classical instructions of the task. In other words, we improved performance by restructuring the environment such that existing skills and tools could have been more effectively applied. Specifically, we restructured the environment through an accurate analysis of how a message is expressed. Taking into account the communicative inferences that the receivers implicitly draw on led to a change in the way the message was processed and in the cognitive skills that participants employed to process it. This, in turn, led participants to think more analytically.

We believe that communication is a form of nudge (Thaler & Sunstein, 2008) in that depending on what we say we can orient another person’s thinking and decision making. Hence, a pragmatic perspective may have important applications in the field of policy interventions, specifically as part of behavior change programs, because they can be used to modify the choice architecture of laypeople (Gigerenzer, Gaissmaier, Kurz-Milcke, Schwartz, & Woloshin, 2007; Hertwig, 2017; Thaler & Sunstein, 2008).
